# Phospho-Akt Immunoreactivity in Prostate Cancer: Relationship to Disease Severity and Outcome, Ki67 and Phosphorylated EGFR Expression

**DOI:** 10.1371/journal.pone.0047994

**Published:** 2012-10-25

**Authors:** Peter Hammarsten, Mariateresa Cipriano, Andreas Josefsson, Pär Stattin, Lars Egevad, Torvald Granfors, Christopher J. Fowler

**Affiliations:** 1 Department of Medical Biosciences, Pathology, Umeå University, Umeå, Sweden; 2 Department of Pharmacology and Clinical Neuroscience, Pharmacology, Umeå University, Umeå, Sweden; 3 Department of Surgical and Perioperative Sciences, Urology and Andrology, Umeå University, Umeå, Sweden; 4 Department of Pathology and Cytology, Karolinska University Hospital, Stockholm, Sweden; 5 Department of Urology, Central Hospital, Västerås, Sweden; University of Nebraska Medical Center, United States of America

## Abstract

**Background:**

In the present study, we have investigated the prognostic usefulness of phosphorylated Akt immunoreactivity (pAkt-IR) in prostate cancer using a well-characterised tissue microarray from men who had undergone transurethral resection due to lower urinary tract symptoms.

**Methodology/Principal Findings:**

pAkt-IR in prostate epithelial and tumour cells was assessed using a monoclonal anti-pAkt (Ser^473^) antibody. Immunoreactive intensity was determined for 282 (tumour) and 240 (non-mlignant tissue) cases. Tumour pAkt-IR scores correlated with Gleason score, tumour Ki67-IR (a marker of cell proliferation) and tumour phosphorylated epidermal growth factor receptor (pEGFR)-IR. For cases followed with expectancy, a high tumour pAkt-IR was associated with a poor disease-specific survival, and the prognostic information provided by this biomarker was additive to that provided by either (but not both) tumour pEFGR-IR or Ki67-IR. Upon division of the cases with respect to their Gleason scores, the prognostic value of pAkt-IR was seen for patients with Gleason score 8–10, but not for patients with Gleason score 6–7.

**Conclusions/Significance:**

Tumour pAkt-IR is associated with both disease severity and disease-specific survival. However, its clinical use as a biomarker is limited, since it does not provide prognostic information in patients with Gleason scores 6–7.

## Introduction

According to recent statistics, the global incidence of prostate cancer (Pca) in 2008 was approximately 900 000 cases [1]. The treatment of Pca varies according to tumour stage and histological grade. Options range from watchful waiting in elderly patients with low-grade disease to oncological treatment of generalised cancer. Radical prostatectomy and radiotherapy are associated with considerable morbidity [2] and a large proportion of the patients would have died of other causes than prostate cancer even without the curative treatment [3]–[5]. Therefore, robust biomarkers are greatly needed to aid treatment decisions.

The serine-threonine kinase Akt is an important regulator of cell proliferation and apoptosis. Downstream effects of Akt activation include the phosphorylation and thereby inactivation of the protein BAD, a pro-apoptotic protein [6] and a change in the transcriptional activity of androgen receptors [7]. Overexpression of a consitutively active form of Akt results in an increased growth of LNCaP prostate cancer cells in a xenograft model [8]. In man, immunohistochemical levels of phosphorylated Akt (pAkt) are higher in prostate tumour tissue and in bone metastases than in non-malignant prostate tissues [9], [10] and are higher in Gleason score 8–10 tumours than lower Gleason scores [11]. Three independent studies have reported that high tumour pAkt immunoreactivitiy is associated with a poor clinical outcome (biochemical relapse assessed with serum prostate-specific antigen (PSA) [9], [12], and survival [13]). In contrast, a fourth study found little prognostic value in pAkt IR [14]. The studies measuring survival, however used rather small cohort sizes (53 and 68 for [14] and [13], respectively). It is therefore important to assess whether or not pAkt immunoreactivity has prognostic significance upon disease-specific survival in a large cohort of well-characterised cases. In addition, it is important to assess whether pAkt immunoreactivity in benign tissue adjacent to tumours has prognostic usefulness, or whether it is restricted to tumour expression alone.

Epidermal growth factor receptor (EGFR) is a cell surface receptor tyrosine kinase responsive to a number of growth factors such as epidermal growth factor and amphiregulin. Phosphorylation of EGFRs leads to activation of a number of different intracellular signalling pathways, in turn resulting in cell growth and survival [15]. Inhibition of EGFR has been shown to enhance castration-induced prostate involution [16]. In Pca, tumour pEGFR immunoreactivity is associated with biochemical recurrence rates [17] and with disease-specific survival [18]. One of the signalling pathways utilised by EGFR is the phosphoinositide 3-kinase/Akt pathway [19] which in Pca cell lines contributes to cell migration mediated by EGF and to the transformation of these cells to give them characteristics reminiscent of epithelial-mesenchymal transition [20]. In Pca tumour tissues, pAkt is frequently co-expressed with EGFR [10], but it is not known whether or not cases with a high expression of pEGFR and pAkt may show different clinical outcomes to those with, for example, a high expression of pEGFR but a low expression of pAkt (or vice versa).

In the present study, we investigated pAkt immunoreactivity in a well-characterised Pca tissue array [3], and determined a) the relationship between pAkt immunoreactivity with disease severity and outcome (disease-specific survival) and b) the influence of pEGFR immunoreactivity upon this relationship.

## Methods

### Patients

The formalin-fixed, paraffin-embedded samples used in the present study were collected between 1975 and 1991 at the Central Hospital, Västerås, Sweden, from a total of 412 patients diagnosed with prostate cancer at transurethral resection for lower urinary tract symtoms [3].

The material was collected according to Swedish Regulations at a time when informed consent was not required. The research ethical committee at Umeå university hospital (Regional Ethical Review Board in Umeå, Sweden) approved of the study and waived the need for informed consent. In the database used for the analyses, the tissue samples were given a case number and year, and the patient names were not indicated in the database.

The presence of metastases was determined by bone scans shortly after the transurethral resection, and the patients were followed until death or until 2003. The Gleason scores, the percentage of the specimen that contained tumour and the disease stage were assessed in each sample. Cause of death was determined by evaluation of medical records. Tissue microarrays using cores with a diameter of 0.6 mm were constructed using a Beecher Instrument (Sun Prairie, WI, USA). Each tissue microarray slide (56 in total, usually with cores from 8 cases per slide) contained up to eight (usually five) samples of tumour tissue (which included both primary and secondary Gleason grade areas) and up to four samples of non-malignant tissue from each patient [3].

### Immunohistochemistry

Sections were deparaffinized, rehydrated and thereafter placed in hydrogen peroxide and methanol in 20 min. The sections are then placed in citrate buffer pH 6.0. After boiling for 60 min in a pressure cooker, the samples were placed in TBS buffer and followed by protein block for 15 min (DAKO, Stockholm, Sweden), after which they were exposed to the primary (rabbit anti-phospho-Akt (Ser^473^) monoclonal antibody (736E11, Cell Signaling, Danvers, MA; dilution 1/100) and the secondary system (CSA-kit which includes DAB; K1500, DAKO). Ki67 was analysed either as the number of positive stained cells crossing 11 horizontal lines across the core, or as the percentage of cells positive for this marker (reported in [21]). In the present paper, we have presented the data using the number rather than the percentage of positive stained cells. Unless otherwise stated, very similar results were seen with both methods of scoring. pEGFR (range of scores 0–5), and PDFRß (range of scores 0–3) immunoreactive (IR) scores were available in the database, and have been reported elsewhere [18], [22].

### Analysis of Data

**Figure 1:**
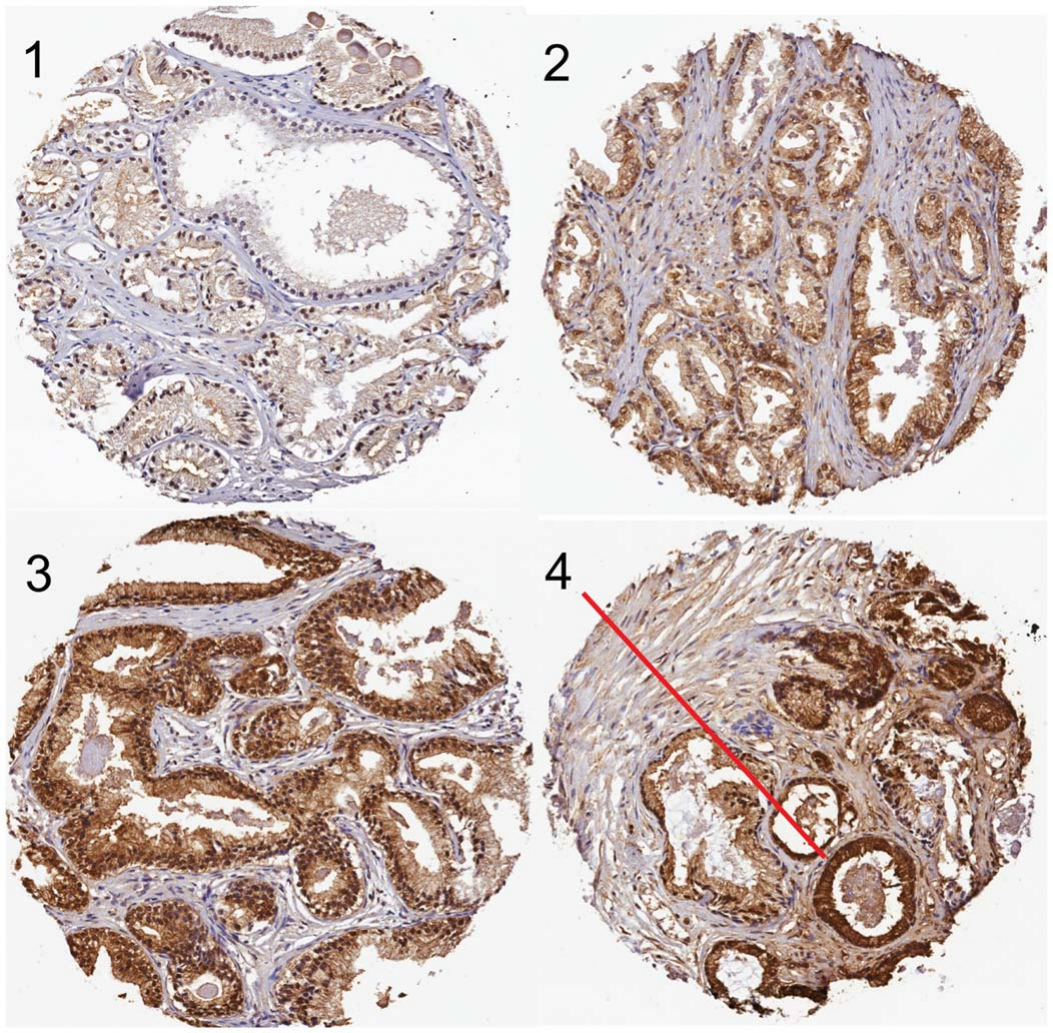
Examples of staining intensities for pAkt-IR ranging from 1, 2, 3 and an area (indicated) with staining 4. These photographs (20×) were used as standards by both evaluators throughout the scoring phase of the project. The top two cores are from tumours, whilst the bottom two cores are from non-malignant tissue.

Tumour and non-malignant cores were scored for pAKT in prostate epithelial and tumour cells from digitally scanned images by two independent evaluators (MC & CF) who did not access the clinical data at the time of evaluation. The scanned scores were analysed on the basis of intensity (0 = no staining, 4 = maximal staining) and distribution (0, 25, 50, 75 or 100% for each intensity). The composite score for each core was then determined. Thus, for example, a core with 25% intensity 3 and 75% intensity 2 would be given a score of 0.25×3+0.75×2 = 2.25. Examples of staining intensities 1–4 are shown in Fig. 1. Not all cores are as well defined as those shown in Figure 1, and in some cases it is a judgement call as to whether or not a core should be scored (such as, for example, a case with mainly stroma and only one or two small areas of epithelial cells). In consequence, we only used cores that had been scored by both evaluators. For the 1648 cores scored independently by both evaluators, an intra-class correlation analysis using a mixed model and testing for consistency gave a Chronbach’s alpha of 0.92. In fact 1219 of the 1648 cores (74%) were scored by the two investigators within 0.5 points of each other. Given that cores with staining somewhere between the units used are not uncommon, a difference in scores of up to 1 can be accepted. A total of 68 (4%) of the cores had differences in scores>1, and these were in consequence rescored, again independently and without accessing the previous scores, given that such large differences could be due to typographical errors or patterns of immunoreactivity that were difficult to score. Following the rescoring, 7 cores were discarded due to poor quality, 55 cores now had scores within 1 unit of each other, and only six cores with score differences>1 remained. These six cores were excluded from the analysis. After exclusion of data from one slide (24 cases) where there were no cores with scores>1, suggesting a technical failure, the median scores for each case were determined, and the medians from the two investigators were averaged and entered into the database. Subsequent investigation of discarded cases in the database indicated that 20 of the 24 cases had Gleason scores 4–5, and 4 had Gleason scores 8–10. The preponderance of Gleason score 4–5 means that their inclusion could bias the prognostic evaluation of pAkt if these immunoreactivities are artifactual. Thus, by excluding them, we are erring on the side of caution.

### Statistics

With the exception of the intraclass correlation coefficient and Cox proportional-hazards regression analyses, which were conducted using SPSS software (SPSS Inc., Chicago, IL, USA), all statistical calculations were undertaken using the statistical package built into the GraphPad Prism 5 computer programme for the Macintosh (GraphPad Software Inc., San Diego, CA, USA). Partial correlation coefficients were calculated on an Excel spreadsheet from the Spearman correlation coefficients for matched samples (i.e. scored for all three parameters under investigation). For survival analyses, an event was defined as death due to prostate cancer and entered into the database as “event = 1″, thereby allowing us to determine disease-specific survival. Death from other causes was censored, as were cases where the patient was alive at the date of last follow-up. Three cases where the disease outcome was not known were excluded from the survival analyses.

## Results

### Distribution of pAkt Immunoreactivity (pAkt-IR) in Non-malignant and Tumour Tissue

Cores from 282 (tumour samples) and 240 (non-malignant tissue samples) cases were scored for pAkt-IR. Consistent with previous studies [9], [10], [12], pAkt-IR was associated with epithelial cells rather than the stroma (Fig. 1). There was a wide range of staining intensities (see e.g. Fig. 1 for examples and Fig. S1 for a distribution curve). In general, the pAkt-IR was greater in the tumour samples than in the non-malignant samples: the median scores were 2.75 and 2.0625 for the tumour and non-malignant samples, respectively. For the 189 cases shown as part of Fig. 1B where both tumour and non-malignant cores were scored, the pAkt-IR scores were significantly correlated (Spearman’s rho = 0.31, P<0.0001), and the scores for the tumour cores were significantly higher than those for the non-malignant cores (P<0.0001, Wilcoxon matched-pairs signed rank test).

### Correlation of pAkt-IR with Disease Severity at Diagnosis

Correlation coefficients for the tumour and non-malignant pAkt-IR scores vs. clinical and biochemical parameters scored for the same samples are summarised in Table. 1. The tumour pAkt-IR correlated significantly with the Gleason score, the percentage of the core that was tumour associated (%ca), the tumour stage, and the tumour Ki67-IR (a measure of cell proliferation). Thus, the tumour pAkt-IR is highly associated with disease severity at diagnosis. The non-malignant pAkt-IR was more weakly associated with these parameters.

**Table 1:** Correlation coefficients for pAkt-IR scores with clinical parameters and with the proliferation marker Ki-67.

	pAkt-IR (T)			pAkt-IR (N)		
Parameter	Sp ρ	P	n	Sp ρ	P	n
Age	−0.03	>0.5	282	−0.12	0.065	240
Gleason score	0.39	<0.0001	282	0.13	0.04	240
% ca^a^	0.27	<0.0001	282	0.17	0.0069	240
Tumour stage	0.30	<0.0001	280	0.14	0.03	238
Ki-67 IR (T)	0.38	<0.0001	279	0.06	>0.3	232
Ki-67 IR (N)	0.03	>0.6	263	0.10	>0.1	236

Sp ρ refers to Spearman’s rho value for the non-parametric comparisons.

a % of core that was tumour associated.

An important question to be investigated is whether the associations between non-malignant pAkt-IR and the clinical markers of disease severity are true correlations, or simply reflect the correlation between tumour and non-malignant pAkt-IR. This can be assessed using the formula:
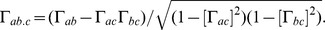
where Γ_ab_ is the Spearman correlation coefficient for the interaction between parameters a and b, and Γ_ab.c_ refers to the first order partial coefficient between parameters a and b when parameter c is taken into consideration. The equation represents the general formula for partial correlation derived in [23]. Using this formula for the samples scored for both tumour and non-malignant pAkt, it becomes clear that the correlations between the non-malignant (N) pAkt-IR and the clinical variables are not significant when the influence of the tumour (T) pAkt-IR is taken into account (Table S1). In contrast, the associations between pAkt-IR (T) and the Gleason score or the tumour proliferation marker Ki67-IR remain significant when either the influence of pAkt-IR (N) or of other clinical variables are taken into account. However, the correlation between pAkt-IR (T) and either the %ca or the tumour stage was lost when the Gleason scores were taken into account (Table S1), although the correlation between pAkt-IR (T) and %ca remained significant when Ki67 was scored as % of cells positive for this marker rather than the number of positive stained cells crossing 11 horizontal lines across the core (data not shown). Thus, it can be concluded that pAkt-IR (T) is primarily associated with the Gleason score and the rate of tumour proliferation as assessed by Ki67-IR, whereas the pAkt-IR (N) is not associated with disease severity.

### Correlation between pAkt-IR and pEGFR-IR

pAkt-IR (T) was significantly correlated to pEGFR-IR scores for both tumour and non-malignant samples (Table 2). In contrast, there were no significant correlations between the pAkt-IR scores and either the stromal or epithelial PDFRß-IR scores (Table 2). The correlation between pAkt-IR (T) and pEGFR-IR (T) remained significant when controlled for pAkt-IR (N) whereas the the correlation between pAkt-IR (T) and either pEGFR-IR in the luminal or basal non-malignant tissue was lost when controlled this variable. Conversely, the correlation between pEGFR-IR (T) and pAkt-IR (N) was lost when controlled for pAkt-IR (T) (Table S2). In other words, the association of pEGFR-IR and pAkt-IR is primarily within the same region of the tissue.

**Table 2:** Correlation coefficients for pAkt-IR scores with pEGFR-IR, total EGFR-IR and PDFRß-IR scores in the tumour and non-malignant tissue samples.

	pAkt-IR (T)			pAkt-IR (N)		
Parameter	Sp ρ	P	n	Sp ρ	P	n
pEGFR-IR (T)	0.27	<0.0001	227	0.17	0.02	193
pEGFR-IR (Nl)	0.24	0.0004	219	0.31	<0.0001	196
pEGFR-IR (Nb)	0.22	0.0008	219	0.30	<0.0001	196
PDFRß-IR (T, ep)	−0.004	0.95	230	−0.0001	1	190
PDFRß-IR (N, ep)	−0.05	0.46	212	0.09	0.23	194
PDFRß-IR (T, st)	0.12	0.06	226	−0.09	0.24	187
PDFRß-IR (N, st)	0.05	0.39	257	0.10	0.12	230

Abbreviations: T, tumour; N, non-malignant; Nl, non-malignant lumiunal, Nb; non-malignant basal; ep, epithelial; st, stroma. Sp ρ refers to Spearman’s rho value for the non-parametric comparisons.

The samples were grouped into four groups on the basis of their tumour pAkt-IR and pEGFR-IR scores and their Gleason scores, incidence of metastases at diagnosis and tumour Ki67-IR scores were compared (Table 3). The distribution of Gleason scores was significantly different for the groups. At the extremes, only 10% of the cases with “low” pEGFR-IR (<3.2) and pAkt-IR (<2.75) scores were diagnosed with Gleason scores in the range 8–10, whereas the corresponding number for cases with “high” pEGFR-IR (≥3.2) and pAkt-IR (≥2.75) scores was 57%. For the Ki67-IR scores, there was no effect of pEGFR-IR at a given pAkt-IR, whereas the scores were higher for pAkt-IR ≥2.75 compared with <2.75 for either pEGFR-IR group. The converse was seen for the incidence of metastases at diagnosis.

**Table 3:** Age, Gleason scores, incidence of metastases at diagnosis and tumour Ki67-IR at diagnosis for the cases divided on the basis of tumour pAkt-IR and pEGFR-IR scores.

			pAkt-IR <2.75		pAkt-IR ≥2.75				
			pEGFR-IR <3.2	pEGFR-IR ≥3.2	pEGFR-IR <3.2	pEGFR-IR ≥3.2			P value
Age in years: median (range) [n]			75 (58–87)[50]	73 (52–88)[50]	76 (64–89)[37]	74 (51–95)[90]			NS^a^
Number (%^b^) of cases with:									
*Gleason*	4–5		19 (38%)	11 (22%)	2 (5%)	5 (6%)			<0.0001^c^
*score*	6		19 (38%)	8 (16%)	19 (51%)	16 (18%)			
	7		7 (14%)	14 (28%)	9 (24%)	18 (20%)			
	8–10		5 (10%)	17 (34%)	7 (19%)	51 (57%)			
pEGFR effect:			P<0.005^c^		P<0.0005^c^				
Number (%^b^) of cases with:									
*Metastases*		No	37 (100%)	35 (88%)	23 (88%)			57 (76%)	<0.01^c^
*at diagnosis*		Yes	0 (0%)	5 (13%)	3 (12%)			18 (24%)	
pEGFR effect:			P<0.05^c^		P<0.05^c^				
Ki67-IR (T) median (range) [n]			0.8 (0–14.4)[50]	1.5 (0–40.8)[50]	1.4 (0–65.1)[36]		2.9 (0–78.5)[88]		<0.0001^a^
pEGFR effect:			NS^d^		NS^d^				

a Kruskal-Wallis test.

b The % value refers to the % of cases for the pAkt-IR/pEGFR-IR group in question (i.e. vertical numbers add up to 100%).

c ķ^2^ test.

d Dunn’s Multiple Comparison Test following significant Kruskal-Wallis test. ^NS^Not significant (P>0.05). "pEGFR effect" refers to the comparison between pEGFR-IR <3.2 and ≥3.2 for the given pAkt-IR tranche. For comparisons between pAkt-IR <2.75 and ≥2.75 for pEGFF <3.2 alone, the significance levels for Gleason score, metastases at diagnosis and Ki67-IR (T) were P<0.01^c^, NS^c^ and P<0.05^d^, respectively. The corresponding significance levels for pEGFR-IR ≥3.2 alone were P<0.01^c^, NS^c^ and P<0.01^d^, respectively.

### Association of pAkt with Disease-specific Survival

For the 282 cases where tumour pAkt-immunoreactivity (pAkt-IR) could be scored, 207 were followed by expectancy, as was the standard approach at the time in Sweden. The other patients received radiotherapy, hormonal treatment or radical prostatectomy. Three cases where outcome was not known were excluded, giving a total of 204 cases available for survival analysis. The corresponding number of cases for the non-malignant pAkt-IR was 194. These samples allow determination as to whether pAkt-IR has prognostic value. For the 204 tumour cases, 101, 20 and 83 cases were below, equal to and above the median value (2.75), respectively.

**Figure 2:**
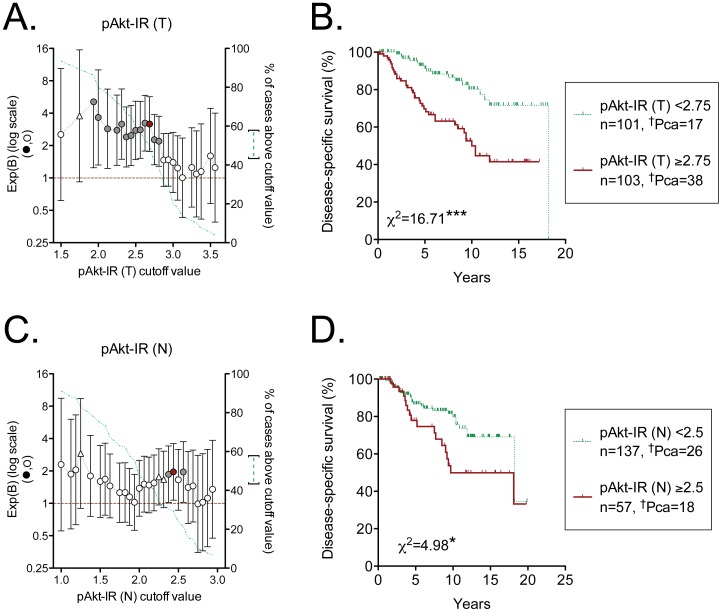
Prognostic significance of tumour and non-malignant pAkt-IR for cases who were followed by expectancy. Panels A and B are for tumour pAkt-IR (n = 204), C and D for non-malignant pAkt-IR (n = 194). In Panels A and C, Exp(B) (±95% confidence intervals), obtained from Cox proportional-hazards regression analyses are shown for different cut-offs. Exp(B) is defined as the increase in risk for death due to prostate cancer for a score above the cut-off value relative to a score below the cut-off value. When both confidence limits are above unity (filled symbols in the figure), the cut-off value provides significant prognostic information. Values with a significance level 0.05<P<0.1 are shown as open triangles. The cut-off value with the highest significance is shown as a red filled symbol. The blue dotted line indicates the % of cases above the cut-off value. Thus, for example, for the symbol in Panel A at pAkt-IR cut-off value 2.5 (i.e sample divided as ≤2.5 and >2.5), 82 cases (40%) were ≤ the cut-off value and 122 cases (60%) above the cut-off value. In Panels B and D, Kaplan-Meier plots are shown for the cut-offs showing the highest significances. ^†^Pca refers to the number of patients who died as a result of their prostate cancer during the follow-up period. The ķ^2^ values are for the log-rank (Mantel-Cox) tests, with the P values shown: ***P<0.001, *P<0.05.

In the search of potential prognostic markers, the choice of cut-off is important. In order to investigate this, Cox proportional-hazards regression analyses were undertaken for both tumour and non-malignant pAkt-IR and over a wide range of cut-off values. The data are shown in Fig. 2. For tumour pAkt-IR, there is a reasonably broad band of cut-off values giving a significant increase in relative risk for values above the cut-off compared to those below the cut-off (Fig. 2A). In other words, dividing the data as, for example, ≤2 and >2 will give a significant prognostic value of pAkt-IR, but so will dividing the data as, for example ≤2.81 and >2.81, or as a median split. We have chosen ≤2.6875 and >2.6875 [i.e <2.75 and ≥2.75, the median split (see above)] as the cut-off, since this gives the greatest significance (shown as the red symbol in Fig. 2A). Using this cut-off, patients with a pAkt-IR ≥2.75 (“high”) had a significantly poorer prognosis than those with a pAkt-IR <2.75 (“low”) (Fig. 2B). The 15 year rate of disease-specific survival was 42±7% and 72±7% for high and low pAkt-IR scores, respectively.

In contrast to the situation for tumour pAkt-IR, non-malignant pAkt-IR scores had a narrow band where significant prognostic value was seen (Fig. 2C), and the Kaplan-Meier plot using the optimal cut-off showed a lower degree of significance than was seen for the tumour pAkt-IR (Fig. 2D). Furthermore, a bivariate COX regression analysis indicated that the significant effect of pAkt-IR (N) was lost when analysed together with pAkt-IR (T) (Table 4).

**Table 4:** COX proportional-hazards regression analyses for tumour and non-malignant pAkt-IR, tumour pEGFR-IR and Ki-67-IR for patients followed by expectancy.

				95% CI for Exp(B)		
Parameter	Cut-off	n	Exp(B)	Lower	Upper	P
***Univariate***						
pAkt-IR (T)	<2.75	101	1			
	≥2.75	103	3.173	1.766	5.698	0.0001
pAkt-IR (N)	<2.5	137	1			
	≥2.5	57	1.963	1.072	3.596	0.029
pEGFR-IR (T)	<3.2	118	1			
	≥3.2	135	3.590	2.018	6.388	<0.0001
Ki67-IR (T)	<1.5	162	1			
	1.5–2.9	57	3.515	1.853	6.668	0.0001
	≥3	67	6.898	3.851	12.357	<0.0001
***Bivariate***						
pAkt-IR (T)	<2.75	76	1			
	≥2.75	73	2.412	1.163	5.003	0.018
pAkt-IR (N)	<2.5	106	1			
	≥2.5	43	1.776	0.878	3.591	0.11
pAkt-IR (T)	<2.75	85	1			
	≥2.75	100	2.772	1.485	5.175	0.001
pEGFR-IR (T)	<3.2	80	1			
	≥3.2	105	2.967	1.477	5.962	0.002
pAkt-IR (T)	<2.75	101	1			
	≥2.75	101	2.218	1.213	4.053	0.010
Ki67-IR (T)	<1.5	104	1			
	1.5–2.9	45	3.082	1.430	6.644	0.004
	≥3	53	6.050	2.960	12.362	<0.0001
pEGFR-IR (T)	<3.2	116	1			
	≥3.2	133	2.891	1.593	5.245	0.0005
Ki67-IR (T)	<1.5	137	1			
	1.5–2.9	50	2.903	1.499	5.622	0.002
	≥3	62	5.904	3.219	10.831	<0.0001
**Trivariate**						
pAkt-IR (T)	<2.75	85	1			
	≥2.75	98	1.897	0.988	3.642	0.054
pEGFR-IR (T)	<3.2	79	1			
	≥3.2	104	2.105	1.027	4.316	0.042
Ki67-IR (T)	<1.5	93	1			
	1.5–2.9	40	2.599	1.148	5.882	0.022
	≥3	50	5.430	2.529	11.661	<0.0001

### Tumour pAkt-IR and Either Tumour pEGFR-IR or Ki-67-IR Provide Additive Prognostic Information

**Figure 3:**
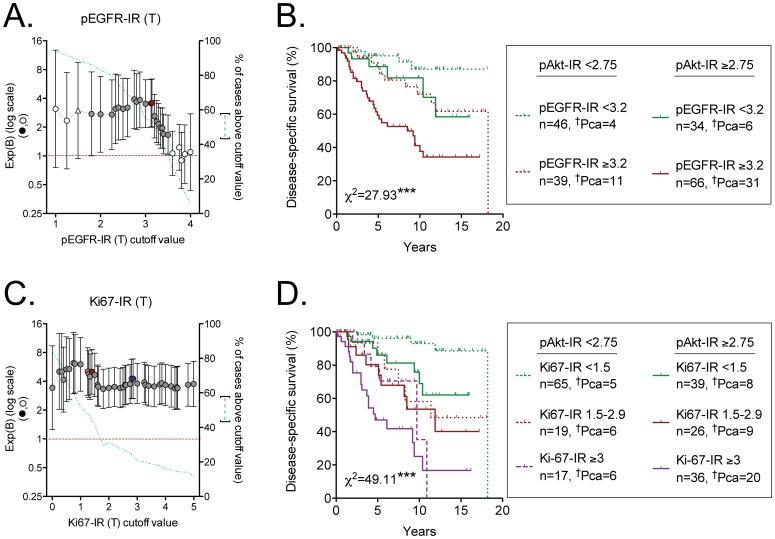
Prognostic significance of tumour pEGFR-IR and Ki67-IR for cases who were followed by expectancy: relationship with tumour pAkt-IR. Panel A shows Exp(B) obtained from Cox proportional-hazards regression analyses are shown for different cut-offs of pEGFR-IR (n = 253). The cut-off value with the highest significance is shown as a red filled symbol. Values with a significance level 0.05<P<0.1 are shown as open triangles. Panel B shows a Kaplan-Meier plot for the 185 cases scored for both tumour pAkt-IR and pEGFR-IR, divided up on the basis of their optimal cut-offs. In Panel C, Exp(B) values are shown for different cut-offs of Ki67-IR (n = 286). The red and blue symbols indicate the highest significance levels for the lower range and for the higher range, respectively. Panel D shows a Kaplan-Meier plot for the 202 cases scored for both tumour pAkt-IR and Ki67-IR, divided up on the basis of their optimal cut-offs. ^†^Pca refers to the number of patients who died as a result of their prostate cancer during the follow-up period. The ķ^2^ values are for the log-rank (Mantel-Cox) tests, with the P values shown: ***P<0.001.

Cox regression data using a wide range of cut-offs were also constructed for the tumor Ki67-IR and pEGFR-IR data available in the database [18], [21]. For pEGFR-IR, the cut-off giving the highest significance corresponded to <3.2 and ≥3.2 (Fig. 3A). In the case of Ki67-IR, there appeared to be two peaks of with approximately equal significance levels, and in consequence the highest points of significance were taken for both peaks, to give three tranches: <1.5, 1.5–2.9 and ≥3 (scores represent the number of tumour cells positive for this biomarker in crossing 11 horizontal lines across the core, Fig. 3C). Using these cut-off ranges, bivariate COX regression analyses indicated that pAkt-IR provided additive prognostic information to that provided either by pEGFR-IR or by Ki67-IR (Table 4). In a tri-variate analysis with all three parameters, the influence of pAkt-IR did not quite reach significance (P = 0.054, Table 4). A similar result was seen when Ki67-IR was expressed as % of cells positive for this marker, although there were fewer patients in the highest tranche (data not shown).

Kaplan-Meier plots of the influence of tumour pAkt-IR upon survival for the entire data set and subdivided on the basis of either the pEGFR-IR or the Ki67-IR cut-offs are shown in Fig. 3B and D. The general pattern seen in both cases was cases with the low scores on both markers had a good prognosis, those with high scores on both markers had a poor prognosis, with the other combinations clustering in the middle with rather similar prognoses. Pairwise comparisons indicated that at a low expression rate of pAkt-IR, the influence of pEGFR-IR does not quite reach significance (and vice versa), whereas at a high expression rate of pAkt-IR, the effect of pEGFR is highly significant (and vice versa) (Table 5).

**Table 5:** Pairwise comparisons of significance levels and 15 year rates of disease specific survival for the combinations of pEGFR-IR and pAkt-IR shown in Fig. 3B.

Comparison	n, †Pca	χ^2^, P^a^	15 year rate of disease-specific survival
pAkt low/pEGFRlow *vs.*	46, 4	χ^2^ = 3.59 P = 0.06	87±6% *vs.*
pAkt low/pEGFRhigh	39, 11		62±10%
pAkt low/pEGFRlow *vs.*	46, 4	χ^2^ = 2.88, P = 0.09	87±6% *vs.*
pAkt high/pEGFRlow	34, 6		58±15%
pAkt high/pEGFRlow *vs.*	34, 6	χ^2^ = 6.67 P<0.01	58±15% *vs.*
pAkt high/pEGFRhigh	66, 31		34±8%
pAkt low/pEGFRhigh *vs.*	39, 11	χ^2^ = 8.43 P<0.005	62±10% *vs.*
pAkt high/pEGFRhigh	66, 31		34±8%

“low” and “high” refer to the cut-offs shown in Fig. 3B.

† Pca refers to the number of patients who died as a result of their prostate cancer during the follow-up period.

a Log-rank (Mantel-Cox) Test.

### Prognostic Usefulness of Tumour pAkt-IR at Different Gleason Scores

**Figure 4:**
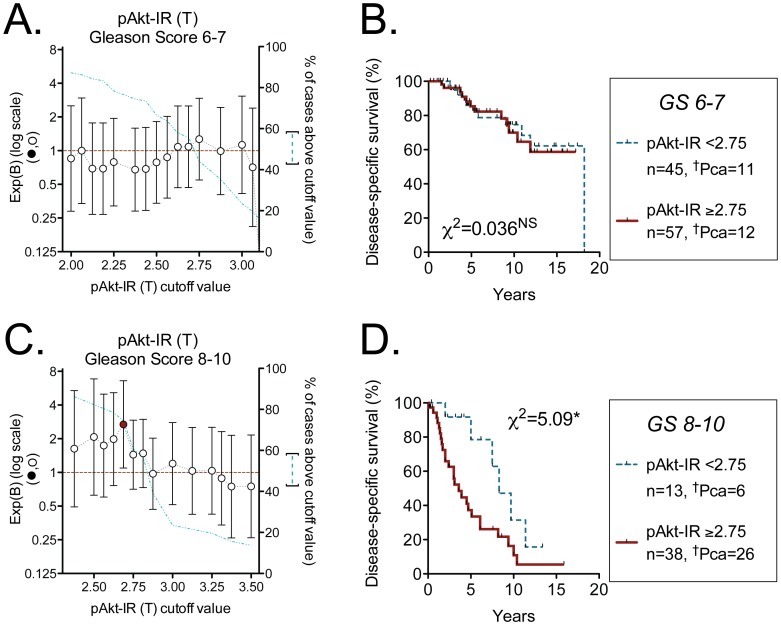
Prognostic significance of tumour and non-malignant pAkt-IR for cases who were followed by expectancy. Panel A and B are for cases with Gleason scores 6–7 (n = 102); Panels C and D are for cases with Gleason scores 8–10 (n = 51). The Exp(B) values obtained from Cox proportional-hazards regression analyses (Panels A and C) and the Kaplan-Meier plots (Panels B and D) were determined as described in the legend to Fig. 2. The ķ^2^ values are for the log-rank (Mantel-Cox) tests, with the P values shown: *P<0.05, ^NS^P>0.8.

The curves shown in Fig. 3 did not take into account the Gleason scores of the samples. There were no cases of Gleason scores 4–5 that were scored for pAkt-IR who died as a result of their cancer, and so only the cases with Gleason scores 6–10 were investigated. A total of 153 cases followed by active expectancy and scored for tumour pAkt-IR had Gleason scores in this range. The Cox regressions at multiple cut-offs again showed an optimal cut-off at tumour pAkt-IR scores of <2.75 and ≥2.75, although the range of significant Exp(B) values was very narrow, with only two cut-off points reaching significance (data not shown). For Gleason scores 6–7 (n = 102), none of the cut-offs reached significance (Fig. 4A) and the Kaplan-Meier plot using the cut-off of <2.75 and ≥2.75 (for illustrative purposes) showed no difference between the survival curves for the two populations (Fig. 4B). At Gleason scores 8–10 (n = 51) only the cut-off at tumour pAkt-IR scores of <2.75 and ≥2.75 was significant (Fig. 4C). From the Kaplan-Meier plot using this cut-off (Fig. 4D), 5 year disease-specific survival rates of 79±14% and 37±9% for pAkt-IR scores of <2.75 and ≥2.75, respectively, were found. For comparative purposes, Exp(B) values were also determined for the Gleason groups 6–7 and 8–10 for both tumour pEGFR-IR and Ki67-IR. pEGFR-IR retained prognostic value for Gleason group 6–7 but not 8–10, whilst Ki67-IR retained prognostic value at both Gleason score groups (Figure S2). A bivariate Cox proportional-hazards regression analysis for the Gleason score 8–10 cases indicated that the tumour pAkt-IR score did not provide additive prognostic information to that provided by the tumour Ki67-IR score (Table S3). When Ki67-IR scores for the Gleason score 8–10 cases were analysed as % of cells positive for this marker rather than the number of positive stained cells crossing 11 horizontal lines across the core, only a single cut-off in the Cox proportional-hazards regression analyses gave a significant Exp(B) value, and the number of cases above this cut-off were so few (n = 5) that bivariate Cox proportional-hazards regression analyses with pAkt-IR were not deemed to be meaningful.

## Discussion

In the present study, pAkt-IR was assessed in a well characterised Pca tissue microarray [3]. There are three main findings, that are discussed in turn below.

### Tumour pAkt-IR is Higher than Non-malignant pAkt-IR and is Correlated to Disease Severity at Diagnosis

Previous studies have reported that pAkt-IR scores are higher in tumour tissue than in non-malignant tissue [9], [10], and the present study has confirmed this finding. In our study, tumour pAkt-IR was correlated to both the Gleason score and the incidence of metastases at diagnosis, whereas the relationship of non-malignant pAkt-IR to disease severity was a reflection of its own correlation with tumour pAkt-IR. Tumour pAkt-IR was also highly correlated to Ki-67 IR, a marker of cell proliferation. Ayala et al. [9] reported in a large cohort that whilst tumour pAkt-IR was greater than the non-malignant pAkt-IR, the scores were correlated with tumour stage but not the Gleason score. In our hands, a significant correlation with tumour stage is also seen, but this is lost when the correlation with Gleason score is taken into account. In smaller cohorts, Le Page et al. [14] did not find any correlation between pAkt-IR and either Gleason score or tumour stage. In contrast, Malik et al. [11] reported that reported that 23/25 cases with Gleason scores 8–10 showed a strong staining intensity for pAkt, whereas the corresponding numbers for Gleason scores 5–6 and 7 were 4/25 and 5/14, respectively. This group also reported a significant correlation between the pAkt staining intensity and the tumour Ki67 labelling index [24]. Thus taken together, there is evidence that tumour pAkt-IR levels are associated with disease severity at diagnosis, but that the degree of association varies between studies. This may be due to differences in the composition of the cohorts. The large cohort described in [9] was primarily composed of cases with Gleason score 6–7 (488 (∼86%) patients of a total 570 scored for pAkt-IR), whereas in our studies, these Gleason scores were less predominant (57 [20%], 121 [43%] and 104 [37%] of the cases had Gleason scores of 4–5, 6–7 and 8–10, respectively).

### Tumour pAkt-IR has Limited Prognostic Usefulness as a Marker for Disease-specific Survival in Pca

In their large cohort, Ayala et al. [9] reported that the 41 cases with a very high pAkt-IR had a poorer biochemical recurrence-free survival (median 97 months) than the remaining 529 cases (median 133 months). The authors also reported that the 8 cases with this cut-off for non-malignant pAkt-IR also had a poorer biochemical recurrence-free survival than the remaining 556 cases. The prognostic value of the tumour pAkt-IR was retained when only cases with Gleason scores 6 or 7 were investigated [9]. In smaller cohorts, both significant and non-significant effects of pAkt-IR upon biochemical recurrence-free survival and/or survival have been reported [12]–[14]. The tissue microarrays of [9], [12], [14] consisted of samples obtained at radical prostatectomy, and whilst the patients had not been treated prior to surgery, it is important to note that the prognostic value of pAkt-IR in these studies has been assessed in this class of patients. In the study of [13], the samples were obtained at either TURP or TRUS-guided biopsy, and the patients were treated during the follow-up period. In contrast, in the present study, the prognostic value of pAkt-IR has been assessed in patients followed by expectancy alone, the long follow-up time allowing assessment of disease-specific survival.

We [16], [25] have previously used receiver operating characteristic (ROC) curves to find optimal cut-off values. Although this method, originally developed to aid detection of radar signals, is widely used to find cut-offs of biochemical markers for disease processes, it may not be strictly valid in cases where the “diagnosis” in question is disease outcome over time rather than the disease itself. Ayala et al. [9] found their cut-off values (at very high pAkt-IR), following “an extensive search for the optimal cut-offs”. We have elected to run Cox proportional-hazards regression analyses over the whole gamut of scores, and pick the cut-off with the highest significance as our choice. This method, although tedious to perform, does take into account the chronological aspect of the endpoint, and additionally shows in a simple manner the range of cut-offs where significant prognostic information is provided. Further, the use of a ROC favours the choice of a single cut-off, which may not always be appropriate: in the present study, the Cox proportional-hazards regression analyses suggest that for tumour Ki67-IR, two cut-offs may be more appropriate.

For a useful prognostic marker, there should be a relatively wide range of cut-off values that provide a significant discrimination of cases with good/poor prognoses. This criteria is simply to allow for application of the marker in clinical praxis, and analysis of the previously published data for tumour pEGFR-IR and Ki67-IR scores [18], [21] indicate that these markers pass this test. For the whole dataset, the tumour pAkt-IR also has a wide range of significant cut-offs, although we did not see any prognostic value at a very high pAkt-IR, in contrast to the study of Ayala et al. [9]. One possible explanation of this difference is in the nature of the samples in their study and in the present study: it may be that biochemical recurrence following radical prostatectomy requires a higher level of pAkt than disease-specific survival in untreated patients. As an aside, it was noted that for both tumour pEGFR-IR and tumour pAkt-IR, the optimal cut-offs measured using Exp(B) as a discrimant were the same as those found using the Youden (optimal) score in 15-year ROC analyses (data not shown and [25]). In the case of non-malignant pAkt-IR, however, the cut-off window is extremely small, and it does not provide additional prognostic value to that seen by tumour pAkt-IR.

Although at first sight the wide window of significant prognostic information is promising, the clinical usefulness of tumour pAkt-IR is limited, since it provides no prognostic information at all at Gleason scores 6–7, i.e. those cases where treatment decisions are the most difficult. The finding, however, that pAkt-IR does provide prognostic information at Gleason scores 8–10 is mechanistically interesting, since it would suggest that activation of this survival pathway adds to the negative prognosis seen in poorly differentiated tumours. Cases with a Gleason score 8–10 in the database have a significantly higher median Ki-67 index than those with lower scores [21]. Although the sample size is small (only 50 cases followed by active expectancy with Gleason scores 8–10 were scored for both tumour pAkt-IR and Ki67-IR), the finding that pAkt-IR does not provide additional prognostic information when Ki67-IR (expressed as the number of positive stained cells crossing 11 horizontal lines across the core) is taken into account (Table S3) is consistent with the suggestion that the prognostic significance of pAkt-IR *per se* in Gleason score cases 8–10 is related to the proliferative capacity of the cells with high pAkt-IR expression.

### Tumour pAkt-IR Correlates with pEGFR-IR and Provides Separate Prognostic Information

In the present study we found that there was a high correlation between tumour pAkt-IR and pEGFR-IR scores, confirming the study of Koumakpayi et al. [17], and consistent both with the finding of a high (54%) co-expression of EGFR and pAkt in prostate tumour cells [10] and the known signalling interrelationship between these two parameters [19]. We have additionally been able to show using partial multiple regression analyses that this correlation is not due to a “third-party” correlation with either the Gleason score or Ki67-IR. In Du145 prostate cancer cells, EGF produces pronounced cell migration and characteristics resembling epithelial-mesenchymal transition in a manner involving activation of Akt [20]. Epithelial-mesenchymal transition-like states are believed to be an important factor in the ability of prostate cancer cells to metastasise [26]. Although the translation of data obtained in cultured cells to the clinical situation is far from easy, these data would predict that cases with high tumour pEGFR-IR and pAkt-IR scores would be more likely to have developed metastases at diagnosis and would be expected to have a poorer prognosis than cases where pEGFR-IR was high but pAkt-IR was low or where both parameters were low. The latter was found to be the case. When the Gleason score was taken into consideration, pEGFR-IR showed prognostic value for cases with Gleason scores 6–7 [10], in contrast to pAkt-IR. Taken together, our data are consistent with the hypothesis that the EGFR – Akt pathway is involved in the severity of the disease, but that additional EGFR pathways play an important role.

A final note concerns the lack of correlation between pAkt-IR and PDGFRß in either the tumour or non-metastatic samples. PDGFRß is a tyrosine kinase implicated in a number of cancers, including prostate cancer [22], [27]. It has long been known that one of the pathways used by PDGFRß is the Akt signalling pathway [28]. The lack of correlation between epithelial PDGFRß-IR and pAkt-IR in the present study would suggest that this pathway is not a dominant pathway of PDGFRß-mediated signalling in the prostate epithelial cells investigated here. It would of course be informative to investigate other receptors that affect growth of Pca cells and which involve Akt signalling in their actions. One such example is the insulin-like growth factor-1 receptor [29]. Similarly, given the finding that a low pERK-IR may compound the influence of pAkt-IR upon biochemical recurrence in Pca [12], it would be informative to investigate this marker in our cohort.

In conclusion, the present study has demonstrated that although tumour pAkt-IR is associated with both disease severity and disease-specific survival, it is of limited clinical usefulness as a biomarker, since it does not provide prognostic information in Gleason 6–7 cases. The association with tumour pEGFR-IR is consistent with the hypothesis that a high activity along the EGFR-Akt signalling pathway facilitates epithelial-mesenchymal transition and thereby an adverse clinical outcome.

## Supporting Information

Figure S1 **Distribution of pAkt-IR scores in tumour (T) and non-malignant (N) samples.** Shown are the scores for 282 (tumour) and 240 (non-malignant tissue) cases, using bin widths of 0.5 IR units to group the samples. The median (with 25% and 75% percentile in brackets) scores were: tumour 2.75 (2.25–3.125) and 2.0625 (1.578–2.609). For the 189 cases scored for both tumour and non-malignant pAkt-IR, the Spearman’s rho value was 0.3105 (P<0.0001), and the median values were significantly different (P<0.0001, Wilcoxon matched-pairs signed rank test).
(TIF)

Figure S2 **Exp(B) values for tumour pEGFR-IR (Panels A and B) and Ki67-IR (Panels C and D) for patients followed by expectancy; influence of Gleason score.** Sample sizes are: pEGFR-IR, Gleason scores 6–7 (A), n = 141; pEGFR-IR, Gleason scores 8–10 (B), n = 59; Ki67-IR, Gleason scores 6–7 (C), n = 140; Ki67-IR, Gleason scores 8–10 (D), n = 61. For explanation of the symbols, see legend to Fig. 3.
(TIF)

Table S1 **Non-parametric partial coefficients for pairwise comparisons of pAkt-IR** ***vs.*** **clinical parameters with a single controlling factor.**
(DOCX)

Table S2 **Non-parametric partial coefficients for pairwise comparisons of pAkt-IR vs. clinical parameters with a single controlling factor.**
(DOCX)

Table S3 **COX proportional-hazards regression analyses for tumour pAkt-IR and Ki-67-IR for patients with Gleason scores 8–10 at diagnosis and followed by expectancy**
(DOCX)
