# Supplementary figures and images for: Phospho-Akt Immunoreactivity in Prostate Cancer: Relationship to Disease Severity and Outcome, Ki67 and Phosphorylated EGFR Expression
Source: PLoS One. 2012 Oct 25;7(10):e47994. doi: 10.1371/journal.pone.0047994 (PMC3485047; doi:10.1371/journal.pone.0047994)

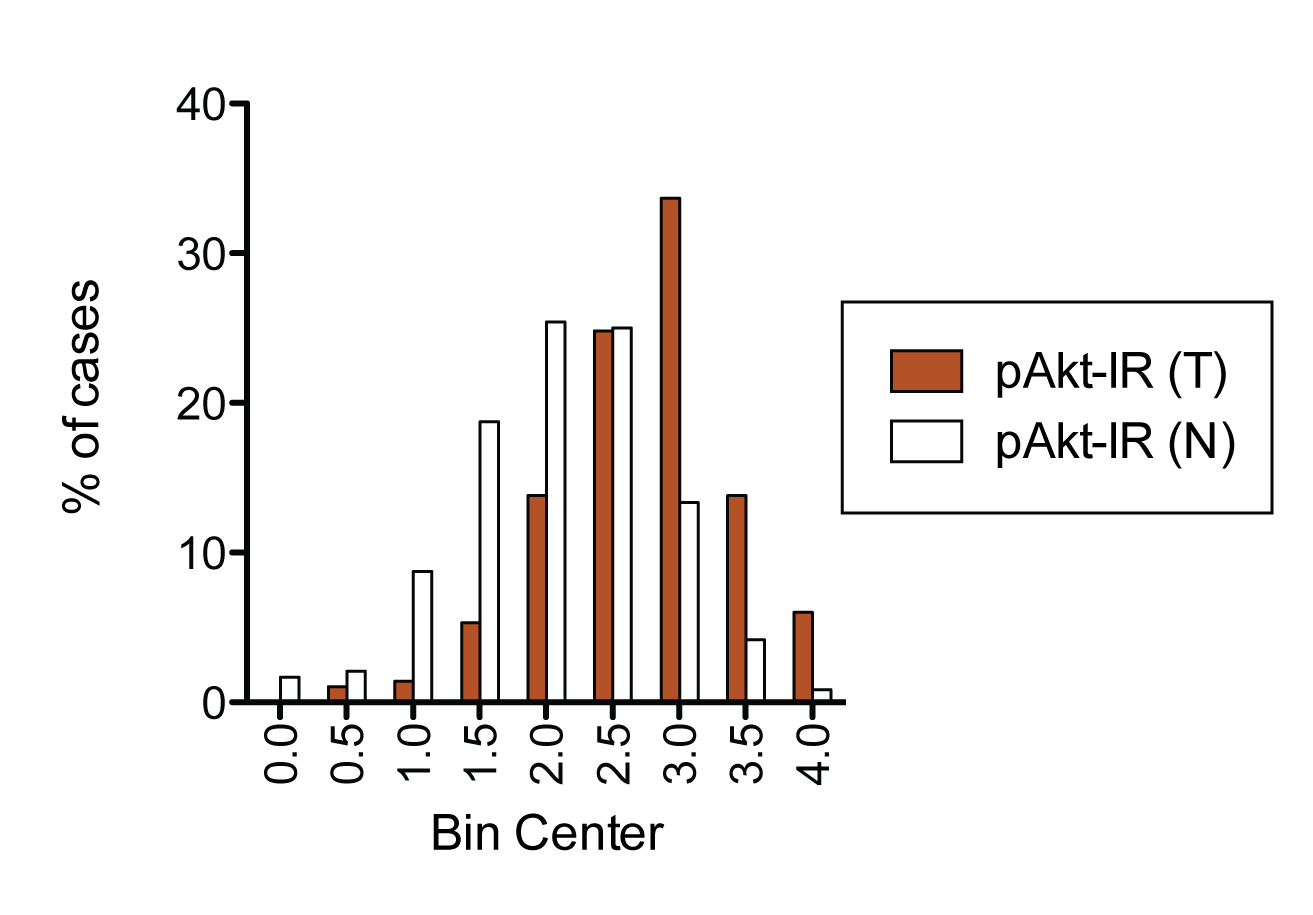

Supplement: Figure S1 — Distribution of pAkt-IR scores in tumour (T) and non-malignant (N) samples. Shown are the scores for 282 (tumour) and 240 (non-malignant tissue) cases, using bin widths of 0.5 IR units to group the samples. The median (with 25% and 75% percentile in brackets) scores were: tumour 2.75 (2.25–3.125) and 2.0625 (1.578–2.609). For the 189 cases scored for both tumour and non-malignant pAkt-IR, the Spearman’s rho value was 0.3105 (P<0.0001), and the median values were significantly different (P<0.0001, Wilcoxon matched-pairs signed rank test). (TIF) [file pone.0047994.s001.tif]

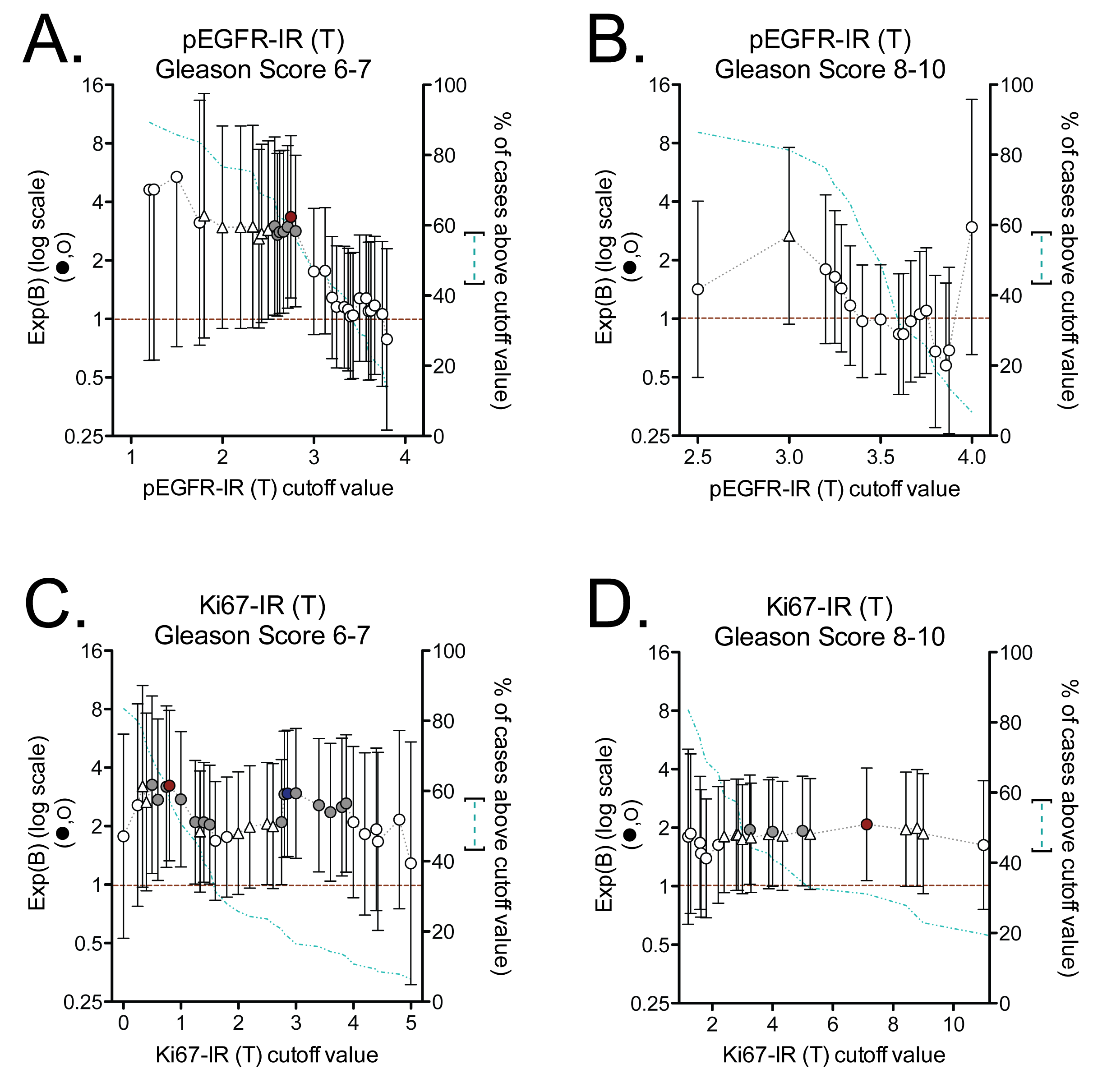

Supplement: Figure S2 — Exp(B) values for tumour pEGFR-IR (Panels A and B) and Ki67-IR (Panels C and D) for patients followed by expectancy; influence of Gleason score. Sample sizes are: pEGFR-IR, Gleason scores 6–7 (A), n = 141; pEGFR-IR, Gleason scores 8–10 (B), n = 59; Ki67-IR, Gleason scores 6–7 (C), n = 140; Ki67-IR, Gleason scores 8–10 (D), n = 61. For explanation of the symbols, see legend to Fig. 3. (TIF) [file pone.0047994.s002.tif]
