# Supplementary material for: Phospho-Akt Immunoreactivity in Prostate Cancer: Relationship to Disease Severity and Outcome, Ki67 and Phosphorylated EGFR Expression
Source: PLoS One. 2012 Oct 25;7(10):e47994. doi: 10.1371/journal.pone.0047994 (PMC3485047; doi:10.1371/journal.pone.0047994)
Supplement: Table S1 — Non-parametric partial coefficients for pairwise comparisons of pAkt-IR vs. clinical parameters with a single controlling factor. (DOCX) [file pone.0047994.s003.docx]

**Supplementary Table S1. Non-parametric partial coefficients for pairwise comparisons of pAkt-IR *vs.* clinical parameters with a single controlling factor**

| **Primary correlation** | **Controlling factor** | **Correlation (threshold value for p<0.05), n** |
| --- | --- | --- |
| **p-Akt (T) *vs.*** |  |  |
| Gleason score | pAkt (N) | **0.337** (P<0.001), n=189 |
|  | Ki67 (T) | **0.243** (P<0.001), n=279 |
|  | % ca^a^ | **0.288** (P<0.001), n=282 |
|  | Tumour stage | **0.277** (P<0.001), n=280 |
|  |  |  |
| Ki67 (T) | pAkt (N) | **0.338** (P<0.001), n=186 |
|  | Gleason score | **0.219** (P<0.001), n=279 |
|  | % ca^a^ | **0.282** (P<0.001), n=279 |
|  | Tumour stage | **0.281** (P<0.001), n=277 |
|  |  |  |
| % ca^a^ | pAkt (N) | **0.207** (P<0.005), n=189 |
|  | Gleason score | 0.027 (NS), n=282 |
|  | Ki67 (T) | 0.104 (NS), n=279 |
|  |  |  |
| Tumour stage | pAkt (N) | **0.209** (P<0.005), n=188 |
|  | Gleason score | 0.111 (NS), n=280 |
|  | Ki67 (T) | **0.157** (P<0.01), n=277 |
|  |  |  |
| **p-Akt (N) *vs.*** |  |  |
| Gleason score | pAkt (T) | 0.026 (NS), n=189 |
| % tumour associated | pAkt (T) | 0.128 (NS), n=189 |
| Tumour stage | pAkt (T) | 0.076 (NS), n=188 |

Correlation coefficients above the threshold value are significant at P<0.05 and are shown in bold type. ^a^% of core that was tumour associated. NS, not significant.
