# Supplementary material for: Phospho-Akt Immunoreactivity in Prostate Cancer: Relationship to Disease Severity and Outcome, Ki67 and Phosphorylated EGFR Expression
Source: PLoS One. 2012 Oct 25;7(10):e47994. doi: 10.1371/journal.pone.0047994 (PMC3485047; doi:10.1371/journal.pone.0047994)
Supplement: Table S2 — Non-parametric partial coefficients for pairwise comparisons of pAkt-IR vs. clinical parameters with a single controlling factor. (DOCX) [file pone.0047994.s004.docx]

**Supplementary Table S2. Non-parametric partial coefficients for pairwise comparisons of pAkt-IR vs. clinical parameters with a single controlling factor**

| **Primary correlation** | **Controlling factor** | **Correlation (threshold value for p<0.05), n** |
| --- | --- | --- |
| **p-Akt (T) *vs.*** |  |  |
| pEGFR (T) | pAkt (N) | **0.223** (P<0.01), n=155 |
|  |  |  |
| pEGFR (Nl) | pAkt (N) | 0.129 (NS), n=155 |
| pEGFR (Nb) | pAkt (N) | 0.127 (NS), n=155 |
|  |  |  |
|  |  |  |
| **p-Akt (N) *vs.*** |  |  |
| pEGFR (T) | pAkt (T) | 0.133 (NS), n=155 |
|  |  |  |
| pEGFR (Nl) | pAkt (T) | **0.274** (P<0.001), n=155 |
|  |  |  |
| pEGFR (Nb) | pAkt (T) | **0.246** (P<0.005), n=155 |
|  |  |  |

Correlation coefficients above the threshold value are significant at P<0.05 and are shown in bold type. NS, not significant.
