# Supplementary material for: Phospho-Akt Immunoreactivity in Prostate Cancer: Relationship to Disease Severity and Outcome, Ki67 and Phosphorylated EGFR Expression
Source: PLoS One. 2012 Oct 25;7(10):e47994. doi: 10.1371/journal.pone.0047994 (PMC3485047; doi:10.1371/journal.pone.0047994)
Supplement: Table S3 — COX proportional-hazards regression analyses for tumour pAkt-IR and Ki-67-IR for patients with Gleason scores 8–10 at diagnosis and followed by expectancy (DOCX) [file pone.0047994.s005.docx]

**Supplementary Table S3. COX proportional-hazards regression analyses for tumour pAkt‑IR and Ki-67-IR for patients with Gleason scores 8-10 at diagnosis and followed by expectancy**

|  |  | |  | |  | |  | |  | |  | |  |
| --- | --- | --- | --- | --- | --- | --- | --- | --- | --- | --- | --- | --- | --- |
|  | |  | |  | |  | |  | |  | |  | |
|  | |  | |  | |  | | 95% CI for Exp(B) | | | |  | |
| Parameter | | Cut-off | | n | | Exp(B) | | Lower | | Upper | | P | |
|  | |  | |  | |  | |  | |  | |  | |
| ***Univariate*** | |  | |  | |  | |  | |  | |  | |
| pAkt-IR (T) | | <2.75 | | 13 | |  | |  | |  | |  | |
|  | | ≥2.75 | | 38 | | 2.686 | | 1.095 | | 6.589 | | 0.03 | |
|  | |  | |  | |  | |  | |  | |  | |
| Ki67-IR (T) | | ≤3.25 | | 33 | |  | |  | |  | |  | |
|  | | 3.28-7.13 | | 9 | | 1.443 | | 0.530 | | 3.932 | | 0.47 | |
|  | | ≥7.2 | | 19 | | 2.227 | | 1.106 | | 4.484 | | 0.02 | |
|  | |  | |  | |  | |  | |  | |  | |
| ***Bivariate*** | |  | |  | |  | |  | |  | |  | |
| pAkt-IR (T) | | <2.75 | | 13 | | 1 | |  | |  | |  | |
|  | | ≥2.75 | | 37 | | 2.024 | | 0.783 | | 5.233 | | 0.15 | |
| Ki67-IR (T) | | ≤3.25 | | 26 | |  | |  | |  | | 0.04 | |
|  | | 3.28-7.13 | | 8 | | 1.629 | | 0.512 | | 5.189 | | 0.41 | |
|  | | ≥7.2 | | 16 | | 2.863 | | 1.263 | | 6.487 | | 0.01 | |
|  | |  | |  | |  | |  | |  | |  | |
